# μ_3_-Oxido-tris­{dichlorido[1,3-bis­(1,3,5-trimethyl­phen­yl)imidazol-2-yl­idene]gold(III)} bis­(trifluoro­methane­sulfon­yl)imide–[bis­(trifluoro­methane­sulfon­yl)imide]­silver(I) (1/2)

**DOI:** 10.1107/S160053681001994X

**Published:** 2010-06-05

**Authors:** Marek Pažický, Frank Rominger, Michael Limbach

**Affiliations:** a CaRLa – Catalysis Research Laboratory, Universität Heidelberg, Im Neuenheimer Feld 584, D-69120 Heidelberg, Germany; b Organisch-Chemisches Institut, Universität Heidelberg, Im Neuenheimer Feld 270, 69120 Heidelberg, Germany

## Abstract

The unusual trinuclear Au^III^ oxide title complex, [Au_3_Cl_6_O(C_21_H_24_N_2_)_3_](C_2_F_6_NO_4_S_2_)·2[Ag(C_2_F_6_NO_4_S_2_)], is the side product of the reaction of [1,3-bis­(1,3,5-trimethyl­phen­yl)imidazol-2-yl­idene]dichloridophenyl­gold(III) with silver bis­(trifluoro­methane­sulfon­yl)imide in the presence of traces of water. In contrast to corresponding Au^I^ complexes, the core structure of the title compound is planar. Two silver(I) bis­(trifluoro­methane­sulfon­yl)imide units are loosely bound to the complex cation. Here the silver atoms, disordered over two positions in a 0.870 (2):0.130 (2) ratio, inter­act either with the lone pairs of three chlorine ligands or two chlorine ligands and one edge of the mesityl π-system. The crystal under investigation was a partial racemic twin.

## Related literature

Tris[(phosphane)gold(I)]oxonium ions are a convenient source for (phosphane)gold cations see: Nesmeyanov *et al.* (1980 ▶). For the trigonal-pyramidal structure of these trinuclear complexes, see, for example: Yang *et al.* (1993 ▶); Schmidbaur *et al.* (1993 ▶); Angermaier & Schmidbaur (1994 ▶, 1995 ▶). For the silver coordination of the bis­(trifluoro­methane­sulfon­yl)imide anion *via* oxygen, see: Nockemann *et al.* (2008 ▶). For details of the preparation, see: Pažický *et al.* (2010 ▶). 
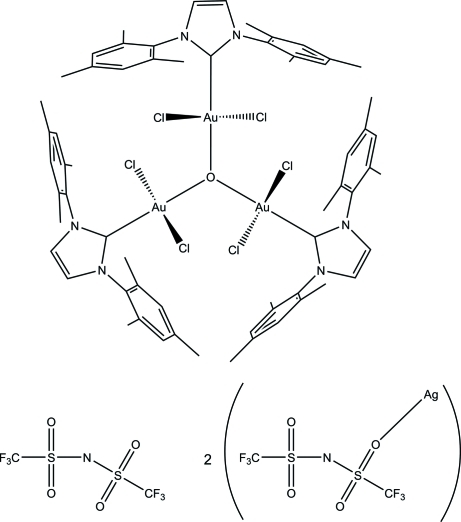


## Experimental

### 

#### Crystal data

[Au_3_Cl_6_O(C_21_H_24_N_2_)_3_](C_2_F_6_NO_4_S_2_)·2[Ag(C_2_F_6_NO_4_S_2_)]
*M* *_r_* = 2789.06
Tetragonal, 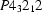

*a* = 13.9472 (9) Å
*c* = 45.724 (3) Å
*V* = 8894.5 (10) Å^3^
*Z* = 4
Mo *K*α radiation
μ = 5.79 mm^−1^
*T* = 200 K
0.25 × 0.17 × 0.11 mm

#### Data collection

Bruker SMART APEX diffractometer
Absorption correction: multi-scan (*SADABS*; Sheldrick, 2008*a* ▶) *T*_min_ = 0.326, *T*_max_ = 0.569
93947 measured reflections
11087 independent reflections
10564 reflections with *I* > 2σ(*I*)
*R*_int_ = 0.068

#### Refinement

*R*[*F* ^2^ > 2σ(*F* ^2^)] = 0.050
*wR*(*F* ^2^) = 0.094
*S* = 1.27
11087 reflections
581 parameters
12 restraints
H-atom parameters constrained
Δρ_max_ = 1.65 e Å^−3^
Δρ_min_ = −1.45 e Å^−3^
Absolute structure: Flack (1983 ▶), 4737 Friedel pairs
Flack parameter: 0.396 (7)

### 

Data collection: *SMART* (Bruker, 2001 ▶); cell refinement: *SAINT* (Bruker, 2001 ▶); data reduction: *SAINT*; program(s) used to solve structure: *SHELXTL* (Sheldrick, 2008*b* ▶); program(s) used to refine structure: *SHELXTL*; molecular graphics: *SHELXTL*; software used to prepare material for publication: *SHELXTL*.

## Supplementary Material

Crystal structure: contains datablocks I, global. DOI: 10.1107/S160053681001994X/hg2690sup1.cif

Structure factors: contains datablocks I. DOI: 10.1107/S160053681001994X/hg2690Isup2.hkl

Additional supplementary materials:  crystallographic information; 3D view; checkCIF report

## Figures and Tables

**Fig. 1.**
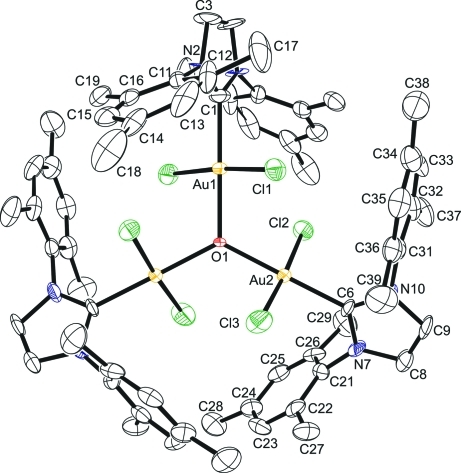
Thermal ellipsoid representation of the trinuclear oxo cation of the title compound. Displacement ellipsoids were plotted at 50% probability level. Hydrogen atoms, the anion and the silver moiety are omitted for the sake of clarity.

**Fig. 2.**
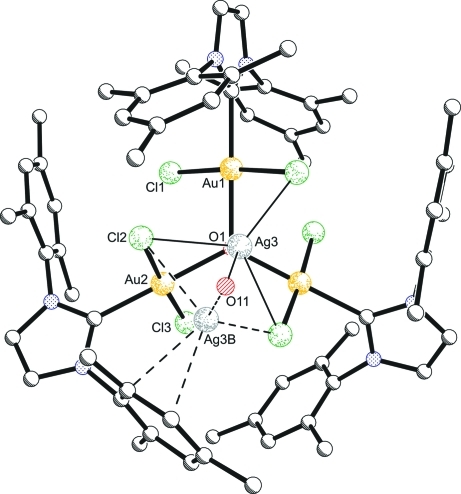
Both alternative silver positions (87% Ag3:13% Ag3B) and their coordination modes to the trinuclear oxo cation in a simple ball and stick representation. From the bis(trifluoromethanesulfonyl)imide anion coordinated to the silver only the coordinating oxygen atom (O11) is shown for clarity. Due to the 2-fold symmetry the same pattern is found at the reverse side of the cation, which is omitted for the sake of clarity as well.

## supplementary crystallographic information

### Comment

The structure of the title compound consists of a trinuclear complex cation around a central oxygen atom with a trigonal planar Au^III^ environment. Loosley bound to this cation are two silver bis(trifluoromethanesulfonyl)imide moieties with a twofold disordered silver position. Finally there is another free bis(trifluoromethanesulfonyl)imide anion to compensate the charge of the cation. Although tris[(phosphane)gold(I)]oxonium ions are a well known source for LAu+ as shown by Nesmeyanov *et al.* (1980), to the best of our knowledge there have been no reports on trimeric [Au_3_O]^+^ complexes with N-heterocyclic carbenes or generally [Au^III^O]^+^ complexes in the literature before.

Both, the trinuclear cation and the free imide anion reside at a special position on a crystallographic twofold axis. The non-crystallographic symmetry of the cation is D_3_. Thus, in contrast to corresponding [(LAu^I^)_3_O]^+^ complexes with pyramidal geometry (*e.g.* Yang *et al.*, 1993; Schmidbaur *et al.*, 1993; Angermaier & Schmidbaur, 1994, 1995), the structure of the Au^III^_3_O core of the title compound is flat. Therefore the Au···Au distances are with 3.4655 (5) and 3.5688 (6) Å necessarily much longer than in the Au^I^ complexes and we assume no aurophilic interactions. The Au—O distances amount to 2.046 (7) and 2.010 (3) Å, the Au—C_NHC_ distances to 2.018 (12) and 1.982 (7) Å. The Cl—Au—Cl axes are inclined with respect to the Au_3_O plane by about 37.0° and 42.0°, which leads to a octahedral chlorine environment for the central oxygen atom with Cl···O distances between 2.986 (5) and 2.996 (5) Å, which is clearly below the van der Waals distance (3.27 Å). As already mentioned two Ag[N(SO_2_CF_3_)_2_] units are loosley bound to this cation. Remarkably there are two alternative positions for the silver center which are occupied by 87% and 13% resp. At the main position the silver is in contact with three chlorine atoms of the cation (Ag···Cl between 2.644 (2) and 2.769 (3) Å), at the alternative position among two chlorine atoms (Ag···Cl 2.302 (8) and 2.754 (8) Å) the silver contacts one edge of the mesityl π-system (Ag···C 2.544 (12) and 2.587 (13) Å). The distorted tetrahedral environment of the silver centers is completed in both cases remarkably with an oxygen atom of the bis(trifluoromethanesulfonyl)imide anion (Ag···O 2.340 (7) and 2.342 (10) Å) rather than the nitrogen. This unexpected coordination mode of the bis(trifluoromethanesulfonyl)imide anion has been observed before by Nockemann *et al.* (2008).

### Experimental

To a solution of phenyldichloro-1,3-bis(1,3,5-trimethylphenyl)imidazol-2-ylidene gold(III) (20 mg, 30.8 µmol) in dry d2-dichloromethane, as described by Pažický *et al.* (2010), silver bis(trifluoromethanesulfonyl)imide (12 mg, 30.8 µmol) was added at room temperature. The solution was stirred for five minutes and filtered over celite. Yellow crystals of the title compound besides white crystals of dichloro-1,3-bis(1,3,5-trimethylphenyl)imidazol-2-ylidene gold(III) bis(trifluoromethanesulfonyl)imide were obtained by slow diffusion of the concentrated filtrate of the crude product into pentane at 8 °C.

### Refinement

Rigid bond restraints were applied to the *U^ij^* values of the nitrogen atom N2 and its surrounding carbon atoms C1, C3, and C11 as well as an isotropic restraint (ISOR) to N2. These prevents atom N2 from going non positive definite. The rest of the adps of the structure was not restrained, as these looked rather reasonable. We can give no convincing explanation for the unsatisfying behavior of N2 and its environment. But in return there is also no plausible possibilty for misinterpretation or atom misassignment here. Obviously the data just results in unrealistic adps in this part of the structure for some reason.

Hydrogen atoms were placed in calculated positions (C–H 0.95– 0.98 Å) and were included in the refinement in the riding model approximation with *U*_iso_(H) set to 1.2–1.5 *U*_eq_(C). A model with staggered conformation with respect to the closest substituent was used for the phenyl-bound methyl groups, which may lead to some ambiguity.

The nature of the disordered silver atom was not clear at first. There were several reasons that led us to the present interpretation. First of all silver bis(trifluoromethanesulfonyl)imide was present in the solution as the only species with an electrophilic atom besides of gold. Furthermore the coordination behaviour to the aromatic π-system and/or to the chlorine ligands as well as to the anion fits fine to the well known geometry of other silver complexes. And finally the refinement of a model with two alternative and partially occupied silver positions converged very well with completely reasonable occupation and displacement parameters.

The refinement of the Flack parameter (Flack, 1983) resulted in a value of 0.396 (7), indicating partial racemic twinning of the crystal.

### Crystal data

**Table:**

[Au_3_Cl_6_O(C_21_H_24_N_2_)_3_](C_2_F_6_NO_4_S_2_)·[Ag(C_2_F_6_NO_4_S_2_)]_2_	*D*_x_ = 2.083 Mg m^−^^3^
*M**_r_* = 2789.06	Mo *K*α radiation, λ = 0.71073 Å
Tetragonal, *P*4_3_2_1_2	Cell parameters from 7947 reflections
Hall symbol: P 4nw 2abw	θ = 2.3–26.5°
*a* = 13.9472 (9) Å	µ = 5.79 mm^−^^1^
*c* = 45.724 (3) Å	*T* = 200 K
*V* = 8894.5 (10) Å^3^	Polyhedron, orange
*Z* = 4	0.25 × 0.17 × 0.11 mm
*F*(000) = 5376	

### Data collection

**Table:**

Bruker SMART APEX diffractometer	11087 independent reflections
Radiation source: fine-focus sealed tube	10564 reflections with *I* > 2σ(*I*)
graphite	*R*_int_ = 0.068
ω scans	θ_max_ = 28.3°, θ_min_ = 2.0°
Absorption correction: multi-scan (*SADABS*; Sheldrick, 2008a)	*h* = −18→18
*T*_min_ = 0.326, *T*_max_ = 0.569	*k* = −18→18
93947 measured reflections	*l* = −61→59

### Refinement

**Table:**

Refinement on *F*^2^	Secondary atom site location: difference Fourier map
Least-squares matrix: full	Hydrogen site location: inferred from neighbouring sites
*R*[*F*^2^ > 2σ(*F*^2^)] = 0.050	H-atom parameters constrained
*wR*(*F*^2^) = 0.094	*w* = 1/[σ^2^(*F*_o_^2^) + (0.0179*P*)^2^ + 57.4255*P*] where *P* = (*F*_o_^2^ + 2*F*_c_^2^)/3
*S* = 1.27	(Δ/σ)_max_ = 0.001
11087 reflections	Δρ_max_ = 1.65 e Å^−^^3^
581 parameters	Δρ_min_ = −1.45 e Å^−^^3^
12 restraints	Absolute structure: Flack (1983), 4737 Friedel pairs
Primary atom site location: structure-invariant direct methods	Flack parameter: 0.396 (7)

### Special details

**Table:**

Geometry. All e.s.d.'s (except the e.s.d. in the dihedral angle between two l.s. planes) are estimated using the full covariance matrix. The cell e.s.d.'s are taken into account individually in the estimation of e.s.d.'s in distances, angles and torsion angles; correlations between e.s.d.'s in cell parameters are only used when they are defined by crystal symmetry. An approximate (isotropic) treatment of cell e.s.d.'s is used for estimating e.s.d.'s involving l.s. planes.
Refinement. Refinement of *F*^2^ against ALL reflections. The weighted *R*-factor *wR* and goodness of fit *S* are based on *F*^2^, conventional *R*-factors *R* are based on *F*, with *F* set to zero for negative *F*^2^. The threshold expression of *F*^2^ > σ(*F*^2^) is used only for calculating *R*-factors(gt) *etc*. and is not relevant to the choice of reflections for refinement. *R*-factors based on *F*^2^ are statistically about twice as large as those based on *F*, and *R*- factors based on ALL data will be even larger.

### Fractional atomic coordinates and isotropic or equivalent isotropic displacement parameters (Å^2^)

**Table:**

	*x*	*y*	*z*	*U*_iso_*/*U*_eq_	Occ. (<1)
Au1	0.47994 (2)	0.47994 (2)	0.0000	0.01855 (9)	
Cl1	0.49234 (17)	0.47738 (18)	0.04966 (4)	0.0362 (5)	
Au2	0.56741 (2)	0.69371 (2)	0.027945 (7)	0.01900 (7)	
Cl2	0.41769 (15)	0.71618 (17)	0.00937 (5)	0.0337 (5)	
Cl3	0.71719 (17)	0.6628 (2)	0.04497 (7)	0.0477 (7)	
O1	0.5837 (3)	0.5837 (3)	0.0000	0.0284 (19)	
Ag3	0.46707 (8)	0.68405 (8)	−0.04559 (2)	0.0536 (3)	0.870 (2)
Ag3B	0.5271 (5)	0.8028 (6)	−0.03324 (15)	0.058 (2)	0.130 (2)
C1	0.3776 (6)	0.3776 (6)	0.0000	0.037 (3)	
N2	0.3920 (5)	0.2874 (5)	0.00768 (19)	0.037 (2)	
C3	0.3046 (7)	0.2407 (7)	0.0040 (3)	0.062 (4)	
H3	0.2933	0.1741	0.0067	0.074*	
C6	0.5503 (6)	0.8017 (6)	0.05561 (17)	0.0222 (16)	
N7	0.5764 (6)	0.8943 (5)	0.05029 (17)	0.0308 (17)	
C8	0.5536 (8)	0.9498 (7)	0.0742 (2)	0.044 (3)	
H8	0.5645	1.0166	0.0763	0.053*	
C9	0.5128 (9)	0.8909 (7)	0.0937 (2)	0.047 (3)	
H9	0.4893	0.9089	0.1124	0.056*	
N10	0.5109 (6)	0.8000 (5)	0.08202 (15)	0.0286 (16)	
C11	0.4835 (7)	0.2484 (6)	0.0171 (2)	0.0332 (19)	
C12	0.4957 (11)	0.2391 (7)	0.0473 (2)	0.058 (4)	
C13	0.5888 (12)	0.2127 (7)	0.0558 (2)	0.062 (4)	
H13	0.6021	0.2046	0.0760	0.075*	
C14	0.6622 (9)	0.1978 (7)	0.0358 (2)	0.045 (3)	
C15	0.6394 (8)	0.1980 (7)	0.0071 (2)	0.037 (2)	
H15	0.6862	0.1773	−0.0066	0.045*	
C16	0.5505 (7)	0.2271 (6)	−0.0033 (2)	0.033 (2)	
C17	0.4194 (14)	0.2516 (10)	0.0696 (3)	0.101 (7)	
H17A	0.3590	0.2686	0.0599	0.152*	
H17B	0.4377	0.3029	0.0832	0.152*	
H17C	0.4110	0.1917	0.0805	0.152*	
C18	0.7632 (11)	0.1742 (9)	0.0464 (3)	0.074 (5)	
H18A	0.8061	0.1674	0.0296	0.112*	
H18B	0.7620	0.1140	0.0575	0.112*	
H18C	0.7864	0.2260	0.0591	0.112*	
C19	0.5314 (7)	0.2320 (7)	−0.0354 (2)	0.037 (2)	
H19A	0.5889	0.2121	−0.0461	0.056*	
H19B	0.5147	0.2978	−0.0409	0.056*	
H19C	0.4781	0.1891	−0.0404	0.056*	
C21	0.6180 (6)	0.9258 (6)	0.02272 (19)	0.0237 (17)	
C22	0.7164 (7)	0.9273 (6)	0.0204 (2)	0.032 (2)	
C23	0.7551 (6)	0.9507 (7)	−0.0071 (2)	0.035 (2)	
H23	0.8228	0.9537	−0.0094	0.042*	
C24	0.6973 (7)	0.9695 (7)	−0.0308 (2)	0.036 (2)	
C25	0.5982 (7)	0.9694 (6)	−0.0267 (2)	0.037 (2)	
H25	0.5579	0.9835	−0.0429	0.044*	
C26	0.5560 (6)	0.9495 (6)	0.0003 (2)	0.0314 (19)	
C27	0.7821 (7)	0.9102 (8)	0.0458 (2)	0.043 (2)	
H27A	0.8488	0.9161	0.0394	0.064*	
H27B	0.7712	0.8456	0.0536	0.064*	
H27C	0.7691	0.9577	0.0611	0.064*	
C28	0.7370 (8)	0.9888 (8)	−0.0611 (2)	0.049 (3)	
H28A	0.6840	1.0019	−0.0746	0.073*	
H28B	0.7727	0.9325	−0.0679	0.073*	
H28C	0.7799	1.0444	−0.0604	0.073*	
C29	0.4533 (8)	0.9624 (8)	0.0046 (3)	0.052 (3)	
H29A	0.4227	0.9762	−0.0143	0.078*	
H29B	0.4423	1.0160	0.0180	0.078*	
H29C	0.4258	0.9037	0.0129	0.078*	
C31	0.4692 (7)	0.7163 (6)	0.09582 (18)	0.0275 (18)	
C32	0.3718 (7)	0.6983 (8)	0.0923 (2)	0.036 (2)	
C33	0.3346 (8)	0.6165 (8)	0.1046 (2)	0.044 (3)	
H33	0.2683	0.6029	0.1023	0.053*	
C34	0.3913 (8)	0.5534 (8)	0.1202 (2)	0.044 (3)	
C35	0.4871 (8)	0.5756 (7)	0.1245 (2)	0.040 (2)	
H35	0.5254	0.5342	0.1362	0.049*	
C36	0.5283 (8)	0.6565 (7)	0.11232 (18)	0.037 (2)	
C37	0.3100 (8)	0.7712 (9)	0.0770 (3)	0.054 (3)	
H37A	0.3500	0.8245	0.0702	0.082*	
H37B	0.2615	0.7954	0.0906	0.082*	
H37C	0.2783	0.7412	0.0602	0.082*	
C38	0.3493 (11)	0.4622 (9)	0.1320 (3)	0.064 (4)	
H38A	0.3986	0.4271	0.1429	0.096*	
H38B	0.3264	0.4226	0.1157	0.096*	
H38C	0.2956	0.4773	0.1450	0.096*	
C39	0.6317 (8)	0.6810 (10)	0.1185 (2)	0.058 (3)	
H39A	0.6606	0.6304	0.1305	0.087*	
H39B	0.6350	0.7421	0.1290	0.087*	
H39C	0.6668	0.6865	0.1000	0.087*	
N11	0.3867 (6)	0.9291 (7)	−0.11355 (19)	0.041 (2)	
S1	0.44103 (18)	0.84305 (18)	−0.09968 (5)	0.0356 (5)	
O11	0.4141 (6)	0.8202 (6)	−0.07076 (16)	0.054 (2)	
O12	0.5421 (5)	0.8391 (6)	−0.10565 (19)	0.056 (2)	
C41	0.3857 (10)	0.7501 (9)	−0.1216 (3)	0.050 (3)	
F1	0.4146 (7)	0.6650 (5)	−0.11142 (19)	0.086 (3)	
F2	0.2925 (6)	0.7518 (5)	−0.12012 (18)	0.070 (2)	
F3	0.4097 (7)	0.7548 (6)	−0.14891 (17)	0.085 (3)	
S2	0.40339 (16)	1.03734 (17)	−0.10456 (5)	0.0305 (5)	
O21	0.4876 (5)	1.0540 (6)	−0.08791 (16)	0.0469 (18)	
O22	0.3869 (6)	1.0968 (5)	−0.12919 (14)	0.0466 (19)	
C42	0.3037 (8)	1.0632 (9)	−0.0801 (2)	0.046 (3)	
F4	0.3092 (6)	1.0118 (6)	−0.05641 (14)	0.085 (3)	
F5	0.2218 (5)	1.0482 (6)	−0.09288 (16)	0.067 (2)	
F6	0.3077 (6)	1.1556 (6)	−0.07284 (16)	0.072 (2)	
N31	0.0200 (6)	0.0200 (6)	0.0000	0.036 (2)	
S3	0.07670 (17)	0.04173 (16)	0.02944 (5)	0.0366 (5)	
O31	0.1221 (6)	0.1333 (5)	0.03190 (16)	0.0480 (18)	
O32	0.0217 (6)	0.0065 (6)	0.05325 (16)	0.059 (2)	
C43	0.1783 (9)	−0.0397 (9)	0.0274 (3)	0.060 (3)	
F7	0.2316 (7)	−0.0298 (7)	0.0511 (2)	0.107 (3)	
F8	0.2302 (5)	−0.0250 (6)	0.00419 (18)	0.078 (2)	
F9	0.1494 (6)	−0.1304 (5)	0.0266 (2)	0.078 (2)	

### Atomic displacement parameters (Å^2^)

**Table:**

	*U*^11^	*U*^22^	*U*^33^	*U*^12^	*U*^13^	*U*^23^
Au1	0.01652 (12)	0.01652 (12)	0.02262 (19)	−0.00444 (16)	0.00103 (12)	−0.00103 (12)
Cl1	0.0411 (13)	0.0417 (12)	0.0257 (9)	−0.0049 (10)	0.0032 (9)	0.0017 (9)
Au2	0.01676 (14)	0.01689 (14)	0.02334 (13)	−0.00161 (11)	0.00300 (12)	−0.00450 (12)
Cl2	0.0203 (10)	0.0392 (13)	0.0416 (12)	0.0038 (8)	−0.0038 (9)	−0.0035 (9)
Cl3	0.0249 (12)	0.0474 (15)	0.0707 (18)	0.0065 (10)	−0.0177 (12)	−0.0156 (13)
O1	0.022 (3)	0.022 (3)	0.042 (5)	−0.015 (3)	0.019 (3)	−0.019 (3)
Ag3	0.0658 (7)	0.0547 (6)	0.0404 (5)	0.0226 (5)	−0.0012 (5)	0.0145 (5)
Ag3B	0.045 (4)	0.078 (5)	0.051 (4)	0.016 (4)	0.004 (3)	0.012 (4)
C1	0.023 (3)	0.023 (3)	0.064 (9)	−0.009 (4)	0.007 (4)	−0.007 (4)
N2	0.026 (3)	0.022 (4)	0.065 (5)	−0.021 (3)	0.014 (3)	0.002 (3)
C3	0.013 (4)	0.032 (5)	0.141 (12)	−0.013 (4)	0.020 (6)	−0.013 (7)
C6	0.026 (4)	0.015 (4)	0.026 (4)	0.004 (3)	−0.001 (3)	−0.010 (3)
N7	0.044 (5)	0.012 (3)	0.036 (4)	−0.008 (3)	0.008 (4)	−0.006 (3)
C8	0.058 (7)	0.023 (5)	0.052 (6)	−0.002 (4)	0.018 (5)	−0.016 (4)
C9	0.070 (8)	0.027 (5)	0.044 (6)	−0.005 (5)	0.023 (6)	−0.024 (4)
N10	0.040 (4)	0.019 (3)	0.027 (3)	0.004 (3)	0.006 (3)	−0.008 (3)
C11	0.045 (5)	0.012 (4)	0.042 (5)	−0.003 (3)	0.024 (4)	0.000 (3)
C12	0.112 (11)	0.021 (5)	0.043 (6)	0.019 (6)	0.044 (7)	0.012 (4)
C13	0.137 (13)	0.014 (5)	0.036 (6)	0.018 (6)	0.005 (7)	0.011 (4)
C14	0.072 (8)	0.015 (4)	0.048 (6)	0.011 (5)	−0.009 (5)	0.001 (4)
C15	0.053 (6)	0.025 (5)	0.033 (5)	−0.010 (5)	0.003 (4)	0.007 (4)
C16	0.047 (6)	0.015 (4)	0.037 (5)	−0.007 (4)	0.010 (4)	−0.001 (4)
C17	0.167 (17)	0.055 (9)	0.081 (10)	0.026 (10)	0.090 (11)	0.022 (7)
C18	0.108 (12)	0.050 (8)	0.065 (8)	0.033 (8)	−0.039 (8)	−0.005 (6)
C19	0.035 (5)	0.027 (5)	0.050 (6)	0.001 (4)	−0.007 (4)	−0.009 (4)
C21	0.018 (4)	0.018 (4)	0.035 (5)	0.005 (3)	0.002 (3)	0.001 (4)
C22	0.039 (5)	0.016 (4)	0.042 (5)	−0.001 (4)	−0.003 (4)	−0.006 (4)
C23	0.019 (4)	0.036 (5)	0.050 (6)	−0.003 (4)	0.003 (4)	0.008 (4)
C24	0.040 (5)	0.029 (5)	0.039 (5)	−0.005 (4)	−0.002 (4)	0.007 (4)
C25	0.044 (6)	0.020 (4)	0.045 (5)	−0.004 (4)	−0.009 (5)	−0.002 (4)
C26	0.026 (4)	0.021 (4)	0.047 (5)	−0.006 (3)	−0.010 (4)	0.001 (4)
C27	0.034 (6)	0.048 (7)	0.046 (6)	−0.012 (4)	−0.010 (5)	0.001 (5)
C28	0.052 (7)	0.046 (7)	0.048 (6)	−0.001 (5)	0.005 (5)	0.019 (5)
C29	0.043 (6)	0.052 (7)	0.062 (7)	0.019 (5)	0.004 (5)	0.014 (6)
C31	0.036 (5)	0.025 (4)	0.022 (4)	0.003 (4)	0.007 (4)	−0.004 (3)
C32	0.030 (5)	0.048 (6)	0.029 (5)	0.014 (4)	0.002 (4)	−0.006 (4)
C33	0.035 (6)	0.066 (8)	0.030 (5)	0.004 (5)	0.014 (4)	0.004 (5)
C34	0.052 (7)	0.051 (7)	0.029 (5)	−0.001 (5)	0.006 (4)	0.000 (4)
C35	0.054 (6)	0.040 (5)	0.027 (5)	0.015 (5)	−0.006 (4)	0.002 (4)
C36	0.049 (6)	0.046 (6)	0.017 (4)	0.002 (5)	−0.001 (4)	−0.003 (4)
C37	0.042 (6)	0.062 (8)	0.060 (7)	0.022 (6)	0.017 (6)	0.007 (6)
C38	0.102 (11)	0.042 (7)	0.047 (7)	−0.006 (7)	0.019 (7)	0.006 (5)
C39	0.053 (7)	0.083 (10)	0.039 (6)	−0.012 (7)	−0.018 (5)	0.004 (6)
N11	0.045 (5)	0.035 (5)	0.044 (5)	0.008 (4)	−0.012 (4)	0.000 (4)
S1	0.0315 (12)	0.0343 (12)	0.0409 (13)	0.0072 (10)	−0.0020 (10)	0.0067 (10)
O11	0.061 (5)	0.056 (5)	0.045 (4)	0.016 (4)	0.000 (4)	0.016 (4)
O12	0.033 (4)	0.058 (5)	0.077 (6)	0.018 (4)	0.009 (4)	0.019 (4)
C41	0.059 (8)	0.043 (7)	0.048 (7)	0.008 (6)	−0.012 (6)	−0.001 (5)
F1	0.116 (7)	0.032 (4)	0.109 (6)	0.016 (4)	−0.023 (6)	0.002 (4)
F2	0.062 (5)	0.058 (5)	0.091 (6)	−0.010 (4)	−0.016 (4)	−0.006 (4)
F3	0.115 (8)	0.086 (6)	0.055 (5)	0.019 (5)	0.004 (5)	−0.017 (4)
S2	0.0290 (11)	0.0361 (13)	0.0264 (11)	0.0080 (9)	0.0001 (9)	0.0017 (9)
O21	0.034 (4)	0.055 (5)	0.052 (4)	0.007 (4)	−0.014 (3)	−0.002 (4)
O22	0.064 (5)	0.049 (5)	0.027 (4)	0.007 (4)	0.003 (3)	0.005 (3)
C42	0.042 (6)	0.064 (7)	0.031 (5)	0.014 (6)	0.004 (5)	0.001 (5)
F4	0.096 (6)	0.115 (7)	0.044 (4)	0.052 (5)	0.033 (4)	0.033 (4)
F5	0.033 (4)	0.086 (6)	0.083 (5)	0.008 (3)	0.008 (3)	0.003 (4)
F6	0.071 (5)	0.069 (5)	0.075 (5)	0.017 (4)	0.012 (4)	−0.025 (4)
N31	0.027 (3)	0.027 (3)	0.055 (7)	0.001 (5)	−0.006 (4)	0.006 (4)
S3	0.0335 (12)	0.0326 (12)	0.0437 (12)	−0.0024 (9)	0.0002 (11)	0.0019 (10)
O31	0.058 (5)	0.033 (4)	0.054 (4)	−0.012 (3)	0.001 (4)	−0.012 (3)
O32	0.064 (5)	0.063 (6)	0.050 (4)	−0.011 (4)	0.021 (4)	0.005 (4)
C43	0.056 (8)	0.053 (8)	0.070 (8)	0.004 (6)	−0.017 (7)	−0.005 (7)
F7	0.101 (7)	0.098 (7)	0.121 (7)	0.028 (6)	−0.078 (6)	−0.002 (6)
F8	0.037 (4)	0.105 (6)	0.091 (6)	0.025 (4)	0.019 (4)	0.019 (5)
F9	0.072 (5)	0.036 (4)	0.124 (7)	0.016 (3)	−0.017 (5)	−0.002 (4)

### Geometric parameters (Å, °)

**Table:**

Au1—C1	2.018 (12)	C22—C27	1.500 (13)
Au1—O1	2.046 (7)	C23—C24	1.376 (13)
Au1—Cl1^i^	2.277 (2)	C23—H23	0.9500
Au1—Cl1	2.278 (2)	C24—C25	1.394 (14)
Cl1—Ag3^i^	2.684 (3)	C24—C28	1.516 (13)
Au2—C6	1.982 (7)	C25—C26	1.394 (14)
Au2—O1	2.010 (3)	C25—H25	0.9500
Au2—Cl3	2.271 (2)	C26—C29	1.457 (14)
Au2—Cl2	2.276 (2)	C27—H27A	0.9800
Au2—Ag3B	3.234 (7)	C27—H27B	0.9800
Cl2—Ag3	2.644 (2)	C27—H27C	0.9800
Cl2—Ag3B	2.754 (8)	C28—H28A	0.9800
Cl3—Ag3B^i^	2.302 (8)	C28—H28B	0.9800
Cl3—Ag3^i^	2.769 (3)	C28—H28C	0.9800
O1—Au2^i^	2.010 (3)	C29—H29A	0.9800
Ag3—O11	2.340 (7)	C29—H29B	0.9800
Ag3—Cl1^i^	2.684 (3)	C29—H29C	0.9800
Ag3—Cl3^i^	2.769 (3)	C31—C32	1.390 (13)
Ag3B—Cl3^i^	2.302 (8)	C31—C36	1.395 (13)
Ag3B—O11	2.342 (10)	C32—C33	1.374 (15)
Ag3B—C25	2.544 (12)	C32—C37	1.504 (14)
Ag3B—C26	2.587 (13)	C33—C34	1.382 (15)
C1—N2	1.322 (10)	C33—H33	0.9500
C1—N2^i^	1.322 (10)	C34—C35	1.385 (15)
N2—C3	1.392 (11)	C34—C38	1.500 (15)
N2—C11	1.452 (13)	C35—C36	1.385 (14)
C3—C3^i^	1.31 (2)	C35—H35	0.9500
C3—H3	0.9500	C36—C39	1.508 (15)
C6—N10	1.327 (10)	C37—H37A	0.9800
C6—N7	1.364 (10)	C37—H37B	0.9800
N7—C8	1.375 (11)	C37—H37C	0.9800
N7—C21	1.455 (11)	C38—H38A	0.9800
C8—C9	1.340 (14)	C38—H38B	0.9800
C8—H8	0.9500	C38—H38C	0.9800
C9—N10	1.376 (11)	C39—H39A	0.9800
C9—H9	0.9500	C39—H39B	0.9800
N10—C31	1.450 (11)	C39—H39C	0.9800
C11—C16	1.352 (12)	N11—S1	1.555 (9)
C11—C12	1.400 (14)	N11—S2	1.582 (9)
C12—C13	1.40 (2)	S1—O11	1.411 (8)
C12—C17	1.484 (16)	S1—O12	1.437 (8)
C13—C14	1.390 (17)	S1—C41	1.812 (13)
C13—H13	0.9500	C41—F3	1.294 (14)
C14—C15	1.349 (14)	C41—F2	1.303 (14)
C14—C18	1.526 (17)	C41—F1	1.338 (13)
C15—C16	1.389 (14)	S2—O22	1.418 (7)
C15—H15	0.9500	S2—O21	1.419 (7)
C16—C19	1.495 (13)	S2—C42	1.821 (10)
C17—H17A	0.9800	C42—F4	1.301 (12)
C17—H17B	0.9800	C42—F5	1.301 (13)
C17—H17C	0.9800	C42—F6	1.332 (14)
C18—H18A	0.9800	N31—S3^i^	1.590 (6)
C18—H18B	0.9800	N31—S3	1.590 (6)
C18—H18C	0.9800	S3—O32	1.420 (7)
C19—H19A	0.9800	S3—O31	1.431 (7)
C19—H19B	0.9800	S3—C43	1.818 (12)
C19—H19C	0.9800	C43—F8	1.302 (15)
C21—C22	1.376 (12)	C43—F7	1.318 (14)
C21—C26	1.383 (12)	C43—F9	1.328 (14)
C22—C23	1.406 (13)		
			
C1—Au1—O1	180.0 (3)	C24—C23—C22	121.5 (9)
C1—Au1—Cl1^i^	92.44 (6)	C24—C23—H23	119.2
O1—Au1—Cl1^i^	87.56 (6)	C22—C23—H23	119.2
C1—Au1—Cl1	92.44 (6)	C23—C24—C25	118.3 (9)
O1—Au1—Cl1	87.56 (6)	C23—C24—C28	122.6 (9)
Cl1^i^—Au1—Cl1	175.12 (12)	C25—C24—C28	119.0 (9)
Au1—Cl1—Ag3^i^	90.42 (7)	C24—C25—C26	122.5 (9)
C6—Au2—O1	179.5 (3)	C24—C25—Ag3B	111.8 (6)
C6—Au2—Cl3	92.1 (2)	C26—C25—Ag3B	76.0 (5)
O1—Au2—Cl3	88.25 (13)	C24—C25—H25	118.7
C6—Au2—Cl2	91.3 (2)	C26—C25—H25	118.7
O1—Au2—Cl2	88.36 (12)	Ag3B—C25—H25	82.3
Cl3—Au2—Cl2	176.55 (9)	C21—C26—C25	116.1 (8)
C6—Au2—Ag3B	100.0 (3)	C21—C26—C29	122.9 (10)
O1—Au2—Ag3B	80.16 (16)	C25—C26—C29	120.7 (9)
Cl3—Au2—Ag3B	123.08 (16)	C21—C26—Ag3B	110.3 (6)
Cl2—Au2—Ag3B	56.83 (15)	C25—C26—Ag3B	72.5 (5)
Au2—Cl2—Ag3	95.30 (8)	C29—C26—Ag3B	91.4 (7)
Au2—Cl2—Ag3B	79.40 (16)	C22—C27—H27A	109.5
Ag3—Cl2—Ag3B	42.07 (18)	C22—C27—H27B	109.5
Au2—Cl3—Ag3B^i^	123.6 (2)	H27A—C27—H27B	109.5
Au2—Cl3—Ag3^i^	92.12 (9)	C22—C27—H27C	109.5
Ag3B^i^—Cl3—Ag3^i^	43.8 (2)	H27A—C27—H27C	109.5
Au2—O1—Au2^i^	125.2 (3)	H27B—C27—H27C	109.5
Au2—O1—Au1	117.41 (17)	C24—C28—H28A	109.5
Au2^i^—O1—Au1	117.41 (17)	C24—C28—H28B	109.5
O11—Ag3—Cl2	104.3 (2)	H28A—C28—H28B	109.5
O11—Ag3—Cl1^i^	142.3 (2)	C24—C28—H28C	109.5
Cl2—Ag3—Cl1^i^	104.40 (8)	H28A—C28—H28C	109.5
O11—Ag3—Cl3^i^	100.4 (2)	H28B—C28—H28C	109.5
Cl2—Ag3—Cl3^i^	102.64 (9)	C26—C29—H29A	109.5
Cl1^i^—Ag3—Cl3^i^	96.55 (8)	C26—C29—H29B	109.5
Cl3^i^—Ag3B—O11	115.9 (4)	H29A—C29—H29B	109.5
Cl3^i^—Ag3B—C25	100.4 (4)	C26—C29—H29C	109.5
O11—Ag3B—C25	104.7 (4)	H29A—C29—H29C	109.5
Cl3^i^—Ag3B—C26	114.8 (4)	H29B—C29—H29C	109.5
O11—Ag3B—C26	117.2 (4)	C32—C31—C36	122.2 (9)
C25—Ag3B—C26	31.5 (3)	C32—C31—N10	119.1 (8)
Cl3^i^—Ag3B—Cl2	113.1 (3)	C36—C31—N10	118.7 (8)
O11—Ag3B—Cl2	101.0 (3)	C33—C32—C31	118.1 (9)
C25—Ag3B—Cl2	122.3 (4)	C33—C32—C37	122.3 (10)
C26—Ag3B—Cl2	90.8 (3)	C31—C32—C37	119.5 (10)
Cl3^i^—Ag3B—Au2	79.3 (2)	C32—C33—C34	121.6 (10)
O11—Ag3B—Au2	143.1 (4)	C32—C33—H33	119.2
C25—Ag3B—Au2	105.1 (3)	C34—C33—H33	119.2
C26—Ag3B—Au2	80.4 (3)	C33—C34—C35	118.9 (10)
Cl2—Ag3B—Au2	43.77 (11)	C33—C34—C38	120.2 (11)
N2—C1—N2^i^	111.0 (11)	C35—C34—C38	120.9 (11)
N2—C1—Au1	124.5 (5)	C36—C35—C34	121.7 (9)
N2^i^—C1—Au1	124.5 (5)	C36—C35—H35	119.2
C1—N2—C3	106.3 (9)	C34—C35—H35	119.2
C1—N2—C11	124.7 (7)	C35—C36—C31	117.4 (10)
C3—N2—C11	129.0 (8)	C35—C36—C39	120.5 (10)
C3^i^—C3—N2	108.1 (6)	C31—C36—C39	122.1 (10)
C3^i^—C3—H3	126.0	C32—C37—H37A	109.5
N2—C3—H3	126.0	C32—C37—H37B	109.5
N10—C6—N7	106.8 (7)	H37A—C37—H37B	109.5
N10—C6—Au2	128.1 (6)	C32—C37—H37C	109.5
N7—C6—Au2	125.0 (6)	H37A—C37—H37C	109.5
C6—N7—C8	109.2 (7)	H37B—C37—H37C	109.5
C6—N7—C21	123.1 (7)	C34—C38—H38A	109.5
C8—N7—C21	127.6 (7)	C34—C38—H38B	109.5
C9—C8—N7	106.5 (8)	H38A—C38—H38B	109.5
C9—C8—H8	126.8	C34—C38—H38C	109.5
N7—C8—H8	126.8	H38A—C38—H38C	109.5
C8—C9—N10	108.3 (8)	H38B—C38—H38C	109.5
C8—C9—H9	125.9	C36—C39—H39A	109.5
N10—C9—H9	125.9	C36—C39—H39B	109.5
C6—N10—C9	109.2 (8)	H39A—C39—H39B	109.5
C6—N10—C31	125.2 (7)	C36—C39—H39C	109.5
C9—N10—C31	125.5 (7)	H39A—C39—H39C	109.5
C16—C11—C12	125.2 (11)	H39B—C39—H39C	109.5
C16—C11—N2	119.0 (9)	S1—N11—S2	124.0 (6)
C12—C11—N2	115.7 (9)	O11—S1—O12	115.5 (5)
C11—C12—C13	114.1 (10)	O11—S1—N11	115.3 (5)
C11—C12—C17	125.5 (14)	O12—S1—N11	115.5 (5)
C13—C12—C17	120.3 (12)	O11—S1—C41	104.1 (6)
C14—C13—C12	122.6 (10)	O12—S1—C41	106.5 (6)
C14—C13—H13	118.7	N11—S1—C41	96.8 (5)
C12—C13—H13	118.7	S1—O11—Ag3	124.1 (5)
C15—C14—C13	117.8 (11)	S1—O11—Ag3B	122.1 (5)
C15—C14—C18	121.9 (11)	Ag3—O11—Ag3B	49.0 (3)
C13—C14—C18	120.1 (11)	F3—C41—F2	108.0 (10)
C14—C15—C16	123.0 (10)	F3—C41—F1	107.7 (10)
C14—C15—H15	118.5	F2—C41—F1	107.4 (11)
C16—C15—H15	118.5	F3—C41—S1	112.8 (10)
C11—C16—C15	116.4 (9)	F2—C41—S1	112.5 (8)
C11—C16—C19	123.0 (10)	F1—C41—S1	108.3 (8)
C15—C16—C19	120.6 (9)	O22—S2—O21	117.7 (5)
C12—C17—H17A	109.5	O22—S2—N11	109.2 (5)
C12—C17—H17B	109.5	O21—S2—N11	114.7 (5)
H17A—C17—H17B	109.5	O22—S2—C42	104.4 (5)
C12—C17—H17C	109.5	O21—S2—C42	105.7 (5)
H17A—C17—H17C	109.5	N11—S2—C42	103.7 (5)
H17B—C17—H17C	109.5	F4—C42—F5	109.7 (11)
C14—C18—H18A	109.5	F4—C42—F6	108.9 (9)
C14—C18—H18B	109.5	F5—C42—F6	107.7 (10)
H18A—C18—H18B	109.5	F4—C42—S2	111.0 (8)
C14—C18—H18C	109.5	F5—C42—S2	111.3 (7)
H18A—C18—H18C	109.5	F6—C42—S2	108.2 (8)
H18B—C18—H18C	109.5	S3^i^—N31—S3	121.8 (7)
C16—C19—H19A	109.5	O32—S3—O31	119.2 (5)
C16—C19—H19B	109.5	O32—S3—N31	108.3 (5)
H19A—C19—H19B	109.5	O31—S3—N31	117.2 (5)
C16—C19—H19C	109.5	O32—S3—C43	104.1 (6)
H19A—C19—H19C	109.5	O31—S3—C43	102.5 (6)
H19B—C19—H19C	109.5	N31—S3—C43	103.0 (4)
C22—C21—C26	124.1 (8)	F8—C43—F7	109.8 (11)
C22—C21—N7	118.1 (8)	F8—C43—F9	107.2 (11)
C26—C21—N7	117.8 (8)	F7—C43—F9	107.1 (11)
C21—C22—C23	117.2 (8)	F8—C43—S3	112.1 (9)
C21—C22—C27	123.0 (9)	F7—C43—S3	109.4 (9)
C23—C22—C27	119.7 (9)	F9—C43—S3	111.1 (9)
			
C1—Au1—Cl1—Ag3^i^	131.82 (6)	N7—C21—C22—C23	175.2 (8)
O1—Au1—Cl1—Ag3^i^	−48.18 (6)	C26—C21—C22—C27	174.1 (9)
Cl1^i^—Au1—Cl1—Ag3^i^	−48.18 (6)	N7—C21—C22—C27	−7.9 (13)
C6—Au2—Cl2—Ag3	140.2 (2)	C21—C22—C23—C24	−1.3 (14)
O1—Au2—Cl2—Ag3	−40.19 (15)	C27—C22—C23—C24	−178.3 (9)
Cl3—Au2—Cl2—Ag3	−50.6 (18)	C22—C23—C24—C25	3.1 (14)
Ag3B—Au2—Cl2—Ag3	39.12 (19)	C22—C23—C24—C28	−176.1 (9)
C6—Au2—Cl2—Ag3B	101.1 (3)	C23—C24—C25—C26	−1.2 (14)
O1—Au2—Cl2—Ag3B	−79.3 (2)	C28—C24—C25—C26	178.1 (9)
Cl3—Au2—Cl2—Ag3B	−89.7 (18)	C23—C24—C25—Ag3B	−88.0 (10)
C6—Au2—Cl3—Ag3B^i^	168.8 (4)	C28—C24—C25—Ag3B	91.2 (10)
O1—Au2—Cl3—Ag3B^i^	−10.8 (3)	Cl3^i^—Ag3B—C25—C24	−1.4 (8)
Cl2—Au2—Cl3—Ag3B^i^	−0.4 (19)	O11—Ag3B—C25—C24	−121.8 (7)
Ag3B—Au2—Cl3—Ag3B^i^	−87.8 (2)	C26—Ag3B—C25—C24	119.8 (10)
C6—Au2—Cl3—Ag3^i^	136.3 (2)	Cl2—Ag3B—C25—C24	124.7 (7)
O1—Au2—Cl3—Ag3^i^	−43.33 (16)	Au2—Ag3B—C25—C24	80.2 (8)
Cl2—Au2—Cl3—Ag3^i^	−32.9 (18)	Cl3^i^—Ag3B—C25—C26	−121.2 (6)
Ag3B—Au2—Cl3—Ag3^i^	−120.4 (2)	O11—Ag3B—C25—C26	118.4 (6)
C6—Au2—O1—Au2^i^	179 (100)	Cl2—Ag3B—C25—C26	4.9 (7)
Cl3—Au2—O1—Au2^i^	−53.29 (8)	Au2—Ag3B—C25—C26	−39.5 (6)
Cl2—Au2—O1—Au2^i^	127.34 (6)	C22—C21—C26—C25	4.6 (13)
Ag3B—Au2—O1—Au2^i^	70.74 (14)	N7—C21—C26—C25	−173.4 (7)
C6—Au2—O1—Au1	−1(30)	C22—C21—C26—C29	−169.7 (9)
Cl3—Au2—O1—Au1	126.71 (8)	N7—C21—C26—C29	12.3 (13)
Cl2—Au2—O1—Au1	−52.66 (6)	C22—C21—C26—Ag3B	84.5 (10)
Ag3B—Au2—O1—Au1	−109.26 (14)	N7—C21—C26—Ag3B	−93.5 (8)
C1—Au1—O1—Au2	45.730 (10)	C24—C25—C26—C21	−2.5 (13)
Cl1^i^—Au1—O1—Au2	132.01 (6)	Ag3B—C25—C26—C21	104.6 (8)
Cl1—Au1—O1—Au2	−47.99 (6)	C24—C25—C26—C29	171.9 (9)
C1—Au1—O1—Au2^i^	−134.270 (10)	Ag3B—C25—C26—C29	−81.0 (9)
Cl1^i^—Au1—O1—Au2^i^	−47.99 (6)	C24—C25—C26—Ag3B	−107.1 (9)
Cl1—Au1—O1—Au2^i^	132.01 (6)	Cl3^i^—Ag3B—C26—C21	−44.0 (8)
Au2—Cl2—Ag3—O11	−125.6 (2)	O11—Ag3B—C26—C21	174.9 (6)
Ag3B—Cl2—Ag3—O11	−57.9 (3)	C25—Ag3B—C26—C21	−112.1 (9)
Au2—Cl2—Ag3—Cl1^i^	79.04 (10)	Cl2—Ag3B—C26—C21	72.0 (6)
Ag3B—Cl2—Ag3—Cl1^i^	146.8 (2)	Au2—Ag3B—C26—C21	29.4 (6)
Au2—Cl2—Ag3—Cl3^i^	−21.24 (9)	Cl3^i^—Ag3B—C26—C25	68.0 (6)
Ag3B—Cl2—Ag3—Cl3^i^	46.5 (2)	O11—Ag3B—C26—C25	−73.1 (7)
Au2—Cl2—Ag3B—Cl3^i^	42.5 (3)	Cl2—Ag3B—C26—C25	−175.9 (6)
Ag3—Cl2—Ag3B—Cl3^i^	−67.9 (3)	Au2—Ag3B—C26—C25	141.4 (6)
Au2—Cl2—Ag3B—O11	167.0 (3)	Cl3^i^—Ag3B—C26—C29	−170.1 (6)
Ag3—Cl2—Ag3B—O11	56.6 (3)	O11—Ag3B—C26—C29	48.8 (8)
Au2—Cl2—Ag3B—C25	−77.7 (4)	C25—Ag3B—C26—C29	121.8 (9)
Ag3—Cl2—Ag3B—C25	172.0 (5)	Cl2—Ag3B—C26—C29	−54.1 (6)
Au2—Cl2—Ag3B—C26	−75.1 (3)	Au2—Ag3B—C26—C29	−96.8 (6)
Ag3—Cl2—Ag3B—C26	174.5 (4)	C6—N10—C31—C32	−91.8 (11)
Ag3—Cl2—Ag3B—Au2	−110.34 (19)	C9—N10—C31—C32	85.8 (12)
C6—Au2—Ag3B—Cl3^i^	134.2 (3)	C6—N10—C31—C36	88.5 (11)
O1—Au2—Ag3B—Cl3^i^	−46.3 (3)	C9—N10—C31—C36	−94.0 (12)
Cl3—Au2—Ag3B—Cl3^i^	35.1 (3)	C36—C31—C32—C33	−2.8 (14)
Cl2—Au2—Ag3B—Cl3^i^	−140.8 (3)	N10—C31—C32—C33	177.5 (8)
C6—Au2—Ag3B—O11	−106.6 (7)	C36—C31—C32—C37	173.3 (9)
O1—Au2—Ag3B—O11	72.9 (6)	N10—C31—C32—C37	−6.5 (13)
Cl3—Au2—Ag3B—O11	154.3 (5)	C31—C32—C33—C34	0.4 (15)
Cl2—Au2—Ag3B—O11	−21.6 (5)	C37—C32—C33—C34	−175.6 (10)
C6—Au2—Ag3B—C25	36.2 (4)	C32—C33—C34—C35	2.6 (15)
O1—Au2—Ag3B—C25	−144.3 (4)	C32—C33—C34—C38	−176.7 (10)
Cl3—Au2—Ag3B—C25	−62.9 (4)	C33—C34—C35—C36	−3.4 (15)
Cl2—Au2—Ag3B—C25	121.2 (4)	C38—C34—C35—C36	175.9 (9)
C6—Au2—Ag3B—C26	16.4 (3)	C34—C35—C36—C31	1.1 (14)
O1—Au2—Ag3B—C26	−164.1 (3)	C34—C35—C36—C39	177.3 (10)
Cl3—Au2—Ag3B—C26	−82.7 (3)	C32—C31—C36—C35	2.0 (13)
Cl2—Au2—Ag3B—C26	101.4 (3)	N10—C31—C36—C35	−178.2 (8)
C6—Au2—Ag3B—Cl2	−85.0 (3)	C32—C31—C36—C39	−174.1 (9)
O1—Au2—Ag3B—Cl2	94.5 (2)	N10—C31—C36—C39	5.7 (13)
Cl3—Au2—Ag3B—Cl2	175.88 (11)	S2—N11—S1—O11	72.3 (8)
O1—Au1—C1—N2	−161.2 (4)	S2—N11—S1—O12	−66.5 (8)
Cl1^i^—Au1—C1—N2	112.5 (5)	S2—N11—S1—C41	−178.5 (7)
Cl1—Au1—C1—N2	−67.5 (5)	O12—S1—O11—Ag3	−49.2 (8)
O1—Au1—C1—N2^i^	18.8 (4)	N11—S1—O11—Ag3	172.0 (5)
Cl1^i^—Au1—C1—N2^i^	−67.5 (5)	C41—S1—O11—Ag3	67.2 (7)
Cl1—Au1—C1—N2^i^	112.5 (5)	O12—S1—O11—Ag3B	10.0 (8)
N2^i^—C1—N2—C3	1.4 (7)	N11—S1—O11—Ag3B	−128.8 (6)
Au1—C1—N2—C3	−178.7 (7)	C41—S1—O11—Ag3B	126.5 (6)
N2^i^—C1—N2—C11	179.6 (10)	Cl2—Ag3—O11—S1	167.3 (5)
Au1—C1—N2—C11	−0.4 (10)	Cl1^i^—Ag3—O11—S1	−54.0 (8)
C1—N2—C3—C3^i^	−3.7 (19)	Cl3^i^—Ag3—O11—S1	61.3 (6)
C11—N2—C3—C3^i^	178.1 (13)	Cl2—Ag3—O11—Ag3B	62.2 (3)
O1—Au2—C6—N10	35 (31)	Cl1^i^—Ag3—O11—Ag3B	−159.2 (3)
Cl3—Au2—C6—N10	−93.2 (8)	Cl3^i^—Ag3—O11—Ag3B	−43.9 (3)
Cl2—Au2—C6—N10	86.2 (8)	Cl3^i^—Ag3B—O11—S1	−43.7 (8)
Ag3B—Au2—C6—N10	142.7 (7)	C25—Ag3B—O11—S1	65.9 (7)
O1—Au2—C6—N7	−144 (30)	C26—Ag3B—O11—S1	97.0 (6)
Cl3—Au2—C6—N7	88.2 (7)	Cl2—Ag3B—O11—S1	−166.3 (5)
Cl2—Au2—C6—N7	−92.4 (7)	Au2—Ag3B—O11—S1	−151.2 (5)
Ag3B—Au2—C6—N7	−35.9 (8)	Cl3^i^—Ag3B—O11—Ag3	65.7 (4)
N10—C6—N7—C8	0.9 (11)	C25—Ag3B—O11—Ag3	175.2 (4)
Au2—C6—N7—C8	179.7 (7)	C26—Ag3B—O11—Ag3	−153.6 (5)
N10—C6—N7—C21	−176.8 (8)	Cl2—Ag3B—O11—Ag3	−56.9 (2)
Au2—C6—N7—C21	2.0 (12)	Au2—Ag3B—O11—Ag3	−41.9 (5)
C6—N7—C8—C9	−0.6 (13)	O11—S1—C41—F3	−174.4 (8)
C21—N7—C8—C9	176.9 (10)	O12—S1—C41—F3	−51.9 (10)
N7—C8—C9—N10	0.2 (14)	N11—S1—C41—F3	67.3 (9)
N7—C6—N10—C9	−0.8 (11)	O11—S1—C41—F2	63.2 (10)
Au2—C6—N10—C9	−179.6 (7)	O12—S1—C41—F2	−174.3 (8)
N7—C6—N10—C31	177.1 (8)	N11—S1—C41—F2	−55.1 (10)
Au2—C6—N10—C31	−1.7 (13)	O11—S1—C41—F1	−55.3 (10)
C8—C9—N10—C6	0.4 (13)	O12—S1—C41—F1	67.2 (10)
C8—C9—N10—C31	−177.5 (9)	N11—S1—C41—F1	−173.6 (9)
C1—N2—C11—C16	−78.3 (11)	S1—N11—S2—O22	149.0 (7)
C3—N2—C11—C16	99.6 (13)	S1—N11—S2—O21	14.5 (9)
C1—N2—C11—C12	99.5 (10)	S1—N11—S2—C42	−100.2 (7)
C3—N2—C11—C12	−82.6 (14)	O22—S2—C42—F4	179.2 (9)
C16—C11—C12—C13	5.3 (15)	O21—S2—C42—F4	−56.1 (10)
N2—C11—C12—C13	−172.4 (8)	N11—S2—C42—F4	64.9 (10)
C16—C11—C12—C17	−172.7 (11)	O22—S2—C42—F5	56.7 (10)
N2—C11—C12—C17	9.7 (16)	O21—S2—C42—F5	−178.6 (8)
C11—C12—C13—C14	0.4 (16)	N11—S2—C42—F5	−57.6 (9)
C17—C12—C13—C14	178.5 (11)	O22—S2—C42—F6	−61.5 (8)
C12—C13—C14—C15	−8.0 (16)	O21—S2—C42—F6	63.3 (8)
C12—C13—C14—C18	176.9 (10)	N11—S2—C42—F6	−175.7 (7)
C13—C14—C15—C16	10.6 (15)	S3^i^—N31—S3—O32	160.1 (4)
C18—C14—C15—C16	−174.3 (10)	S3^i^—N31—S3—O31	21.6 (4)
C12—C11—C16—C15	−3.0 (14)	S3^i^—N31—S3—C43	−90.0 (5)
N2—C11—C16—C15	174.5 (7)	O32—S3—C43—F8	171.7 (9)
C12—C11—C16—C19	175.6 (9)	O31—S3—C43—F8	−63.4 (10)
N2—C11—C16—C19	−6.8 (13)	N31—S3—C43—F8	58.7 (10)
C14—C15—C16—C11	−5.3 (14)	O32—S3—C43—F7	−66.3 (11)
C14—C15—C16—C19	175.9 (9)	O31—S3—C43—F7	58.6 (11)
C6—N7—C21—C22	−93.0 (11)	N31—S3—C43—F7	−179.3 (10)
C8—N7—C21—C22	89.8 (12)	O32—S3—C43—F9	51.8 (11)
C6—N7—C21—C26	85.2 (11)	O31—S3—C43—F9	176.6 (9)
C8—N7—C21—C26	−92.1 (12)	N31—S3—C43—F9	−61.2 (11)
C26—C21—C22—C23	−2.8 (14)		

Symmetry codes: (i) *y*, *x*, −*z*.
